# Regulatory roles of copper metabolism and cuproptosis in human cancers

**DOI:** 10.3389/fonc.2023.1123420

**Published:** 2023-03-23

**Authors:** Zhe Wang, Dekui Jin, Shuaishuai Zhou, Niujing Dong, Yuting Ji, Peng An, Jiaping Wang, Yongting Luo, Junjie Luo

**Affiliations:** ^1^ Department of Nutrition and Health, China Agricultural University, Beijing, China; ^2^ Department of General Practice, The Third Medical Center of Chinese PLA General Hospital, Beijing, China; ^3^ China Astronaut Research and Training Center, Beijing, China

**Keywords:** copper, cancer, copper metabolism, cuproptosis, copper complexes, cancer therapeutics

## Abstract

Copper is an essential micronutrient for human body and plays a vital role in various biological processes including cellular respiration and free radical detoxification. Generally, copper metabolism in the body is in a stable state, and there are specific mechanisms to regulate copper metabolism and maintain copper homeostasis. Dysregulation of copper metabolism may have a great connection with various types of diseases, such as Wilson disease causing copper overload and Menkes disease causing copper deficiency. Cancer presents high mortality rates in the world due to the unlimited proliferation potential, apoptosis escape and immune escape properties to induce organ failure. Copper is thought to have a great connection with cancer, such as elevated levels in cancer tissue and serum. Copper also affects tumor progression by affecting angiogenesis, metastasis and other processes. Notably, cuproptosis is a novel form of cell death that may provide novel targeting strategies for developing cancer therapy. Copper chelators and copper ionophores are two copper coordinating compounds for the treatment of cancer. This review will explore the relationship between copper metabolism and cancers, and clarify copper metabolism and cuproptosis for cancer targeted therapy.

## 1 Introduction

Copper (Cu) is an essential micronutrient for the human body. Copper has four oxidation states: metallic copper, Cu^+^, Cu^2 +^ and Cu^3+^. As a transition metal, copper plays a key role in many biological processes, such as cellular respiration (1), free radical detoxification (2–5), cellular iron metabolism (6), angiogenesis (7), and neurotransmitter synthesis (8). However, excess intracellular copper ions can be toxic to cells (9). The transfer of electrons will occur in the transfer of copper ions with different valence states, resulting in the formation of reactive oxygen species (ROS).ROS can injury biological organic molecules such as proteins, nucleic acids and lipids, and also interfere with the synthesis of iron sulfur clusters to injury countless essential enzymes in cells. In addition, copper overload is seen in Wilson disease (WD), which is a manifestation of dysregulation of organismal copper homeostasis (10). Deficiency of copper ions results in the reduction of multiple enzyme activities, which is also thought to underlie the pathologies of Menkes disease (MD) (11). Therefore, no matter whether copper ions are excessive or deficient, it may be harmful to human body. Copper homeostasis is essential in organisms and dysregulation of copper metabolism leads to the occurrence of some diseases.

Copper is closely related to cancer. It is well known that copper is also involved in tumor formation and progression. Copper levels are elevated in a variety of malignancies, and high levels of copper ions affect tumor proliferation, angiogenesis, as well as metastasis (12–14). In recent years, a novel form of cell death induced by intracellular copper, discovered by Tsvetkov and co-workers (15), which is distinct from oxidative stress-related cell death, is a type of copper-dependent cell death, termed cuproptosis. This review will explore the relationship between copper metabolism, cuproptosis and cancers, providing references for cancer targeted therapy.

## 2 Copper metabolism and cancer

### 2.1 Copper metabolism

In mammals, copper is required for cellular metabolism, but its excess is toxic to cells. Therefore, copper concentration in cells is tightly regulated (16). There are many components involved in cellular copper homeostasis maintenance, including (1) transporters mediating copper absorption, such as copper transporter receptor 1 (Ctr1) (also called SCL1A1), copper transporter receptor 2 (Ctr2), divalent metal transporter 1 (DMT1); (2) enzymes guiding copper ion efflux, such as copper-transporting ATPase 1 (ATP7A) and copper-transporting ATPase 2 (ATP7B); (3) biomolecules that sequester and store copper, such as metallothionein (MT), glutathione (GSH); (4) copper chaperones, such as copper chaperone for superoxide dismutase (CCS), antioxidant protein 1 (Atox1), cytochrome c oxidase copper chaperone 17 (Cox17), which direct copper to copper dependent enzymes and transport copper to organelles that requiring copper (16).

**Figure 1:**
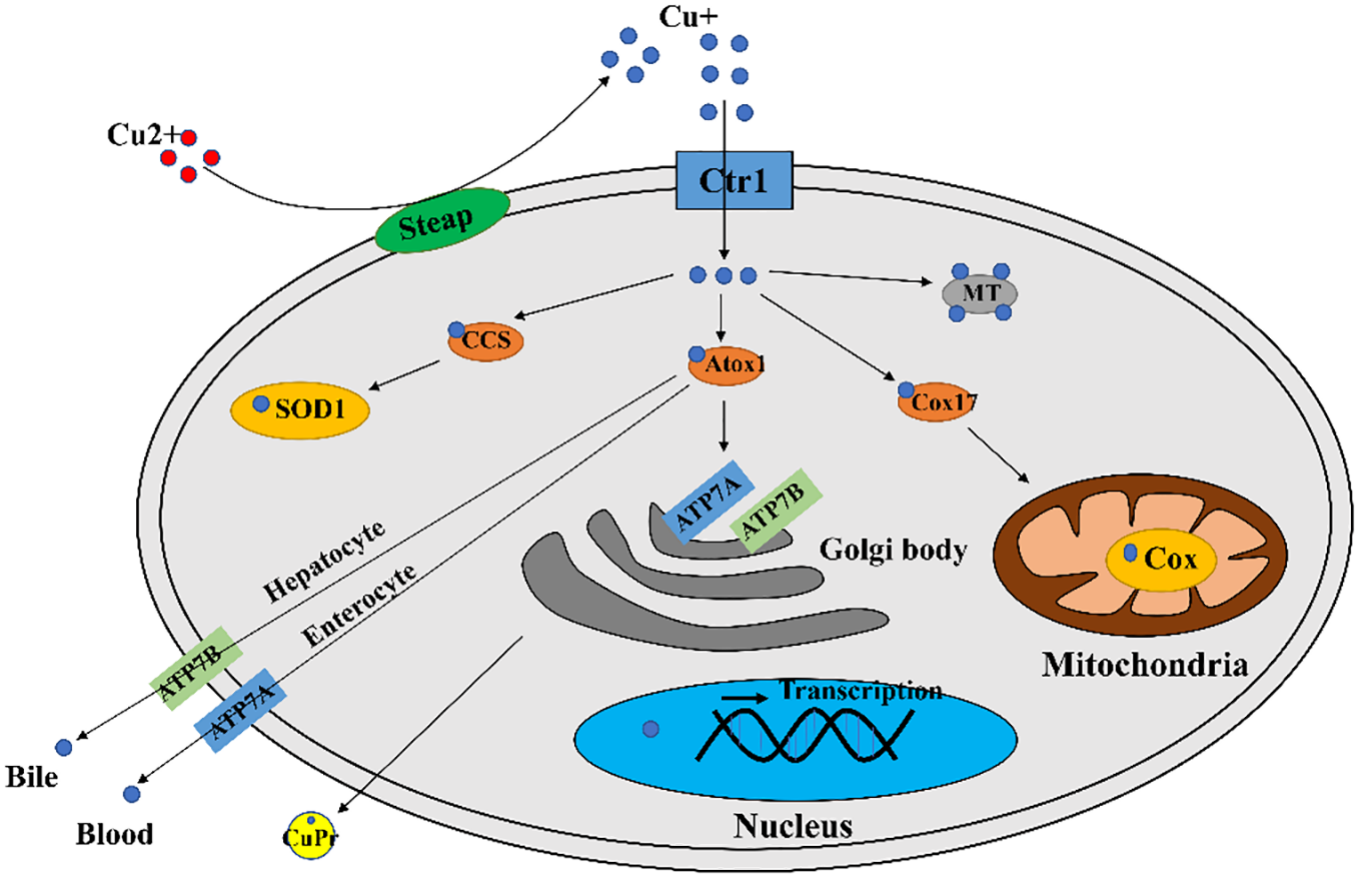
A diagram of cellular copper transport and metabolism. Extracellularly, copper exists as Cu^2+^. The cellular reductases protein family Steap proteins (mainly Steap 2/3/4) reduce Cu^2+^ to Cu^+^, which is transported into the cell *via* Ctr1, and a fraction of Cu^+^ is targeted to cytosolic SOD1 by the copper chaperone CCS to scavenge free radicals. A fraction of Cu^+^ is delivered by the copper chaperone Cox17 to the mitochondrial Cox to generate ATP. A portion of Cu^+^ is delivered to ATP7A/B of the trans Golgi network by the copper chaperone Atox1, which promotes cuproproteins (CuPrs) assembly and secretion. The remaining excess Cu^+^ is sequestered by metallothionein (MT). There are copper sensors in the nucleus that respond to changes in copper concentration through regulating *MT1* and *MT2* gene transcriptions. In enterocytes, ATP7A migrates to the plasma membrane to pump Cu^+^ into the blood. In hepatocytes, ATP7B pumps Cu^+^ into the bile.

A major contributor involved in copper uptake in mammals is Ctr1 (17). It is now generally accepted that Ctr1 transports Cu^+^ in a high affinity manner (18, 19), however in mammalian enterocytes the copper ion is in the form of divalent copper (Cu^2+^), which can be directly transported by divalent metal transporter 1 (DMT1) but cannot be directly utilized by cells (20). Thus enterocytes produced intracellular reductases such as steap2/3/4 to reduce cell surface Cu^2+^ to Cu^+^ and then Cu^+^ can be transported by Ctr1 (21) (**Figure 1**).

Copper ions are transported to specific locations after entering the cell *via* utilization or detoxification pathways, and excess copper will be sequestered by copper storage proteins such as MT and GSH (22). The intracellular trafficking of copper is mediated by copper chaperones, such as CCS, Atox1, Cox17. These copper chaperones assist copper in reaching vital destinations without inflicting damage or becoming trapped in adventitious binding sites (23) In the cytoplasm, CCS mediates Cu^+^ loading and activation of superoxide dismutase 1 (SOD1) (24). The chaperone Cox17 mainly functions to transfer copper to Sco1, Sco2, Cox11 (25), and Sco1/2 plays an important role for cytochrome c oxidase (Cox) formation (26). The role of Atox1 is to bind Cu^+^ and deliver it to P1B type ATPases (27), which includes ATP7A and ATP7B with important roles in the systemic distribution of copper ions.

Excessive copper ion accumulation in cells may generate ROS, causing oxidative damage to cells. Thus, the excess Cu^+^ will be bound by MT or GSH, maintaining the concentration of intracellular Cu ions within a suitable range (16). The copper ion export in mammals is dependent on large multi transmembrane proteins ATP7A and ATP7B (16). There are multiple routes for copper ions transport out of the cell. For example, copper ions in intestinal epithelial cells enter the blood directly *via* ATP7A, and ATP7B in hepatocytes pumps copper ions into the bile (16) (**Figure 1**).

### 2.2 Copper deficiency and copper overload

Copper metabolism is meticulously controlled in living organisms to maintain the level of copper in a reasonable range. Defects in molecules involved in copper metabolism will resultin disturbed copper homeostasis and related diseases. WD and MD are two typical copper disorders resulting from dysregulation of copper metabolism (28).

MD is a copper deficiency disease caused by mutations in *ATP7A* (28). The main function of ATP7A is to transport copper from enterocytes to the blood, where it plays a vital role in intestinal absorption of copper and renal copper reabsorption (29). Defective ATP7A impedes intestinal copper absorption, ultimately leading to severe systemic copper deficiency, as well as deficiency of cuproenzymes in tissues, such as brain. The symptoms exhibited by MD patients include neuropathy, hypopigmentation, seizures, and hypothermia (30, 31). In addition, copper deficiency can lead to impaired energy levels, increased oxidative damage, and changes in immune cell structure and function in living organisms (32).Studies have suggested that copper deficiency may result in a higher frequency of infections as well as a higher risk of cardiovascular disease (33, 34).

WD is a copper overload disease caused by mutations in *ATP7B* (28). The main functions of ATP7B is to transport copper to the trans Golgi to facilitate assembly and secretion of cuproenzymes (29). In addition to hepatocytes, ATP7B also acts as a copper ion exporter, excreting it into bile. Because of gene mutations, ATP7B dysfunction leads to copper ion accumulation in the liver. When the capacity of the liver for storage is exceeded, copper spills into the circulation and subsequently enters and deposits into other tissues (*e.g.* eye and brain), causing oxidative stress that damages the tissues (28). The typical pathological features of WD are neurological abnormalities and acute liver failure (35, 36).

### 2.3 Cancer and copper metabolism

Cancer has long been a research hotspot in the life sciences as well as in medicine. The incidence and mortality rates of cancer have been rising rapidly over the past few decades. It has now become the disease responsible for the largest number of deaths in the population worldwide (37). Among these, lung cancer is the most common and associated with the highest mortality in the population. The second highest incidence was for female breast cancer, followed by prostate and colorectal cancer. The top four cancers in order of mortality were lung, colorectal, stomach and liver cancer (37). Copper ion metabolism is also involved in the progression of these cancers (38).

In fact, copper in the human body has a great association with cancer, and there are a large number of medical studies showing that the serum copper levels in cancer patients, as well as in tumor tissues, can be higher or lower (mostly high) compared with normal individuals (**Tables 1**, **2**). When tumor is removed, serum copper return to comparable levels with healthy individuals (13). In several serum medical detections of breast cancer patients, it was found that copper levels were significantly elevated in the serum of breast cancer patients compared with the healthy population (51). Similarly, elevated levels of copper have been reported in the serum of patients with oral cancer (47), gallbladder cancer (46), liver cancer (49), pancreatic cancer (57), and prostate cancer (61). Serum copper levels were found to be decreased in the serum of patients with certain cancers, such as colorectal cancer (56) and endometrial cancer (68). In colorectal and breast cancer, increased serum copper levels correlated with cancer staging and progression (69). And serum elevated copper levels are also associated with hematological malignancies either in relapse or in disease progression (70). Because of the variation of serum copper levels in cancer, it may be used as an indicator to diagnose certain tumors. The Cu/Zn ratio has been widely recognized as an indicator for the auxiliary diagnosis of tumors, and we summarized most up-to-date evidence of Cu/Zn ratio in the diagnosis of cancers (71, 72).

**Table 1:** Copper level variation in cancer tissues among different cancers.

Cancer types	Copper levels	Mechanism	References
Breast cancer	Elevated	Cu as an effective factor of angiogenesis	(39, 40)
Colorectal cancer	Elevated	Excess Cu damages DNA directly or through ROS.	(41)
Esophageal cancer	Elevated	Not mentioned	(42)
Ovarian cancer	Elevated	Alteration of the relationship between trace elements and decreased catabolism; Increased tumor synthesis of neurofibromin	(43)
Gastric cancer	Elevated	Alteration of the relationship between trace elements and decreased catabolism; Increased tumor synthesis of neurofibromin;High concentrations of Cu damage DNA through toxic hydroxyl radicals	(44, 45)
Gallbladder cancer	Elevated	Copper may be involved in the initial biological insult	(46)

**Table 2:** Copper level variation in the serum of different cancers.

Cancer types	Copper levels	Mechanism	References
Oral cancer	Elevated	Increase of oxidation process; Changes of ceruloplasmin activity	(47, 48)
Gallbladder cancer	Elevated	Malignant cell necrosis leads to copper release into the serum	(46)
Liver cancer	Elevated	Inflammatory processes activate Ceruloplasmin; Hepatocytes are damaged to release copper	(49, 50)
Breast cancer	Elevated	Cu is a cell growth promoting factor for rapid tumor cell growth; Necrosis that arises in tumor tissue occurs through the release of copper into the circulation; Copper causes mutations by damaging DNA through the generation of reactive oxygen species	(51–54)
Esophageal cancer	Elevated	Not mentioned	(55)
Colorectal cancer	Decreased	Unclear	(56)
Pancreatic cancer	Elevated	Not mentioned	(57)
Bladder cancer	Elevated	Copper accumulation has potential toxic effects; Copper is required for angiogenesis and tumor growth factor	(58, 59)
Renal cancer	Elevated	Not mentioned	(60)
Prostatic cancer	Elevated	Copper binds metallothionein with higher affinity, substituting zinc for metallothionein binding	(61)
Thyroid cancer	Elevated	High concentrations of Cu can damage DNA through toxic hydroxyl radicals; Copper as a cofactor in angiogenesis	(45, 62, 63)
Cervical carcinoma	Elevated	Copper damages DNA by generating reactive oxygen species; Copper as a tumor angiogenesis factor	(64, 65)
Lung cancer	Elevated	Ceruloplasmin may be re-catalyzed on the surface of tumor cells or in the peripheral blood in patients with cancer, thereby inhibiting its catabolism	(66, 67)
Endometrial cancer	Decreased	Not mentioned	(68)

An increasing number of existing studies have demonstrated that copper is critical for the development of cancer not only as a component to maintain cell function, but also as a central hub in cell signaling pathways involving cell proliferation, angiogenesis, and metastasis (73). Cu^+^ is redox active and is able to promote the production of ROS and thereby activate tumor signaling, leading to tumor proliferation (69). In part, studies of the relationship between co-binding proteins (or chaperones) and cell proliferation identified that Atox1, a copper dependent transcription factor, promoted the expression of genes encoding cell replication (74). In recent studies, copper has also been found to have a specific role in the mitotic signaling pathway of tumorigenesis. Studies using drosophila and mouse models found that copper uptake *via* Ctr1 activates the mitogen activated protease kinase (MAPK) (75). Among molecules of this pathway, MEK1 is a copper binding protein that, when bound to copper ions, is able to push the MEK1-ERK interaction to promote the phosphorylation of ERK1 and ERK2, ultimately leading to carcinogenesis and promoting tumor growth (76, 77).

Copper is able to induce a number of proangiogenic responses (78), and increases proangiogenic gene expression by stabilizing nuclear hypoxia inducible factor-1 (HIF-1) (79). Copper also activates some angiogenic factors such as basic fibroblast growth factor (bFGF), tumor necrosis factor alpha (TNF-α), IL-1, IL-6 and IL-8 (80). In addition, copper stimulates the proliferation and metastasis of vascular endothelial cells (81). It directly binds to the angiogenic growth factor angiopoietin, enhancing its affinity for endothelial cells (82).

Copper is implicated in epithelial to mesenchymal transition (EMT), which is necessary for cancer metastasis (83). Increasing studies have shown that copper enhances the invasive and metastatic abilities of cancer cells through the activation of metabolic and proliferative enzymes (84, 85). For example, copper is indispensable for the activity of lysine oxidase (LOX) and lysine oxidase like (LOXL) proteins, which catalyze the cross-linking of collagen and elastin in the extracellular matrix (ECM), and create preconditions for tumor development and metastasis (86). It was found that a copper dependent redox protein named memo affected metastasis of breast cancer cells by increasing intracellular ROS levels (87). Recently, the copper chaperone Atox1 has also been shown to be essential in breast cancer cell migration (88, 89).

Copper is redox active and easily interconverts between Cu^+^ and Cu^2+^. Many important enzymes utilize this property of copper to exert their functions in redox reactions in living organisms (69). Because copper is capable of generating excess ROS, copper transporters and chaperones have evolved to regulate copper uptake, efflux, and distribution within cells (33). Dysregulation of copper metabolism may lead to oxidative stress, such as decreased SOD1 activity and increased superoxide anion in different animal models (90, 91). Copper deficiency may also increase oxidative stress in mitochondria by inhibiting cytochrome c oxidase activity (92). And the dysregulation of copper metabolism may cause cancer, as copper deficiency may have effects on the oxidative system (33). Copper is a cofactor of SOD1, and the main function of SOD is to scavenge free radicals to prevent cells from oxidative stress injury, especially playing a crucial role in scavenging ROS generated from mitochondria (93, 94). SOD protein has three isoenzymes in humans. In particular, Cu/Zn SOD (SOD1) is a SOD with a bimetallic enzymatic function, which requires copper to catalyze the reaction and zinc to increase catalytic efficiency and enzyme stability (95–98). Copper deficiency leads to reduced SOD1 activity, and reduced SOD1 activity contributes to carcinogenesis (99). Copper deficiency also alters the activity of other enzymes involved in oxidative stress as well as ROS scavengers (*e.g.* catalase, metallothionein) (33). Alterations in these proteins may cause deregulation of oxidative stress, overproduction of ROS as well as deregulation of oxidative stress in the body may impair DNA repair machinery which is also an important mechanism in cancer development (100).

Ceruloplasmin is involved in copper metabolism, which is the main carrier of copper in the human body, and about 90% of copper in plasma is found in ceruloplasmin. In addition, ceruloplasmin is a multi-copper oxidase that plays an important role in iron homeostasis (101). When Fe^2+^ exported from ferroportin, the sole iron exporter, ceruloplasmin promotes cellular iron export by oxidizing iron ion from Fe^2+^ to Fe^3+^ (102). Although ceruloplasmin synthesis and secretion are not affected by copper levels, copper deficiency may result in decreased ceruloplasmin stability and activity (103). Ceruloplasmin is also closely linked to cancer, and studies have indicated that significant ceruloplasmin gene expression occurs in many tumors and that the overall incidence of cancer is positively correlated with serum ceruloplasmin levels and may be able to serve as a prognostic marker in some cancers (104–107).

## 3 Copper and targeted therapy in cancer

Among current treatments for cancer, targeted therapy is considered to be highly promising because its intervention can be selectively performed on molecules and pathways involved in the growth and developmental progression of tumors (108). Considering copper as a nutrient for cancer growth, angiogenesis, and metastasis, it may be an attractive target in cancer therapy (109). Copper metal binding compounds have great potential in cancer therapy. When copper binding compounds are mentioned, copper chelators and copper ionophores come to mind.Currently, copper chelators and copper ionophores have great potential value in cancer targeted therapy.

Copper chelators are able to bind to copper and reduce its bioavailability, thereby inhibiting angiogenesis and hindering cancer growth and metastasis (110). So far, some copper chelation methods have been used in clinical trials and provided some new strategies for the treatment of cancer (111, 112). Copper chelators with anticancer activity are tetrathiomolybdate (TTM), D-penicillamine (D-Pen) and others. There are studies demonstrating TTM exerts significant efficacy in the treatment of squamous cell carcinoma (113), lung (114), breast (115) and prostate cancer (116). It is important to note, however, that copper chelators are simply anticancer and are not sufficient by themselves to kill malignant cells, therefore, it needs to be combined with other drugs to achieve a therapeutic effect that is promising for cancer (117).

Copper ionophore in contrast to copper chelators,is able to increase intracellular copper bioavailability. There are various modes of action of copper ionophores, such as DNA interaction, proteasome inhibition as well as ROS generation (69). Typical copper ionophores are chloroquinol and disulfiram (DSF), and they can release coordinately available copper in the intracellular reducing environment, increasing the bioavailability of copper inside cells (118). Chloroquinol and DSF are able to cause intracellular production of ROS and inhibit the activity of proteasomes in cancer cells, which enables apoptosis (119). Chloroquinol and disulfiram have also been shown to reduce tumor growth in models of prostate and breast cancer (120–122). Copper chelators are able to inhibit cuproptosis, whereas copper ionophores induce cuproptosis. Cuproptosis, already introduced in a previous text, is a copper dependent cytotoxicity with a unique mechanism leading to cell death (123). However, the field of copper metal binding compounds to treat cancer is still in an early stage of development, and although clinical trials have been conducted and are able to give some strategies to treat cancer, they still need to be explored further to overcome their disadvantages. The lack of selectivity for targeting cancer cells is one of the challenges in this field.

**Figure 2:**
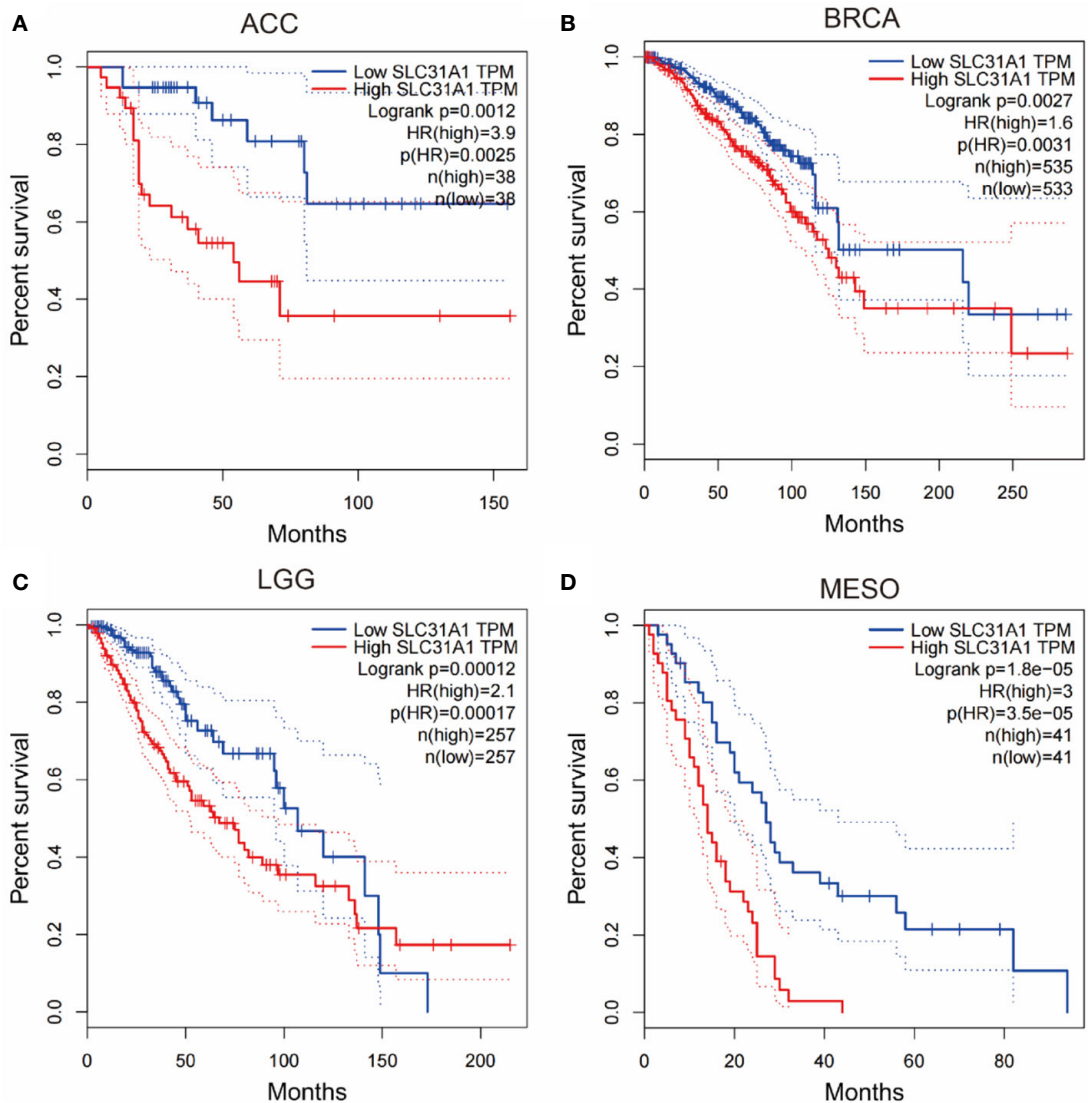
Survival curves for overall survival of high versus low expressing SLC31A1. **(A)** ACC, adrenocortical carcinoma; **(B)** BRCA, breast invasive carcinoma; **(C)** LGG, brain lower grade glioma; **(D)** MESO, mesothelioma. The overall survival rate of low expression of SLC31A1was significantly higher than that of high expression of SLC31A1. SLC31A1, solute carrier family 31 member 1 (copper ion transporter); HR, hazard rate. (http://gepia.cancer-pku.cn/index.html; Accessed 10 October 2022).

In addition, inhibiting the expression of copper transporters may provide some reference for cancer therapy. We searched through the gepia database and analyzed for survival curves between the expression levels of the copper ion transporter SLC31A1 and cancer patient survival. We found that the expression level of copper importer in cancer tissue may have a close relationship with patient survival. Analysis of the survival curves between the expression levels of SLC31A1 and cancer patient survival showed that lower SLC31A1 expression significantly increased overall survival compared with individuals with higher SLC31A1 expression in Adrenocortical carcinoma (ACC), Breast invasive carcinoma (BRCA), Brain Lower Grade Glioma (LGG), Mesothelioma (MESO), Skin Cutaneous Melanoma (SKCM) (**Figure 2**). The inverse association between SLC31A1 expression and patient survival suggests that excessive copper ion uptake may promote cancer progression and increase patient mortality. This correlation may provide a potential mechanism for developing novel cancer therapies through inhibition SLC31A1 expression or removal of large amounts of copper ions in tumor tissues.

## 4 Cuproptosis

Recently, a novel mode of cell death was discovered by Tsvetkov et al. (15). It is a copper-dependent, regulated, distinct from other known cell death regulatory mechanisms, and this copper-dependent manner of death has been termed “cuproptosis”. Heavy metal overload such as iron will cause deleterious effect on cells. An example is ferroptosis, an iron-dependent form of cell death caused by unrestricted lipid peroxidation (124). Cuproptosis results from mitochondrial stress. Copper can directly bind to ester acylated components of the tricarboxylic acid cycle, with subsequent aggregation of copper bound lipidated mitochondrial enzymes and loss of iron sulfur protein clusters, finally leading to the occurrence of cuproptosis (15).

**Figure 3:**
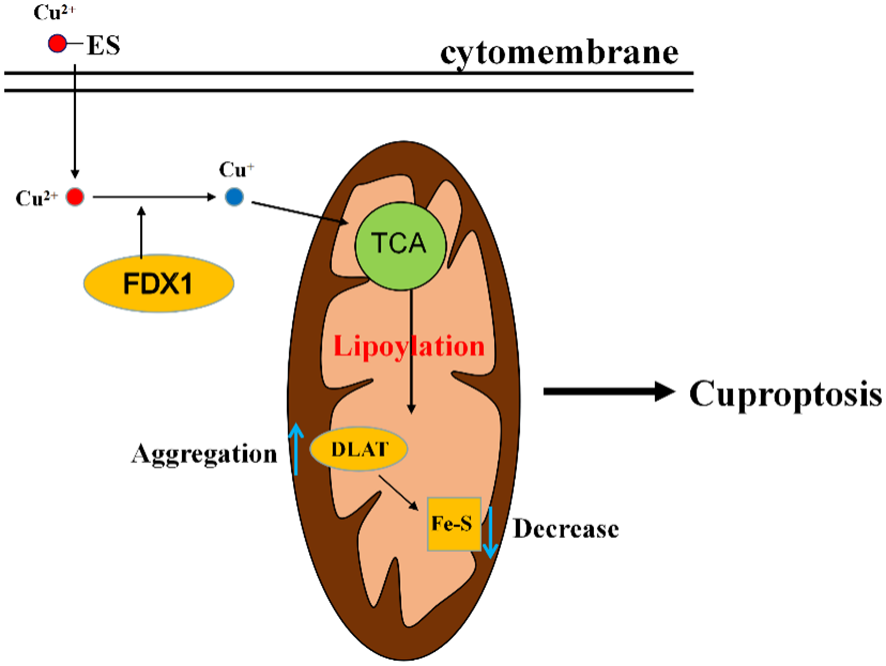
A diagram of the simple mechanism of cuproptosis. Elesclomol imports Cu^2+^ into the cell, and then reduced to Cu^+^ by FDX1. Cu^+^ binds to lipoylated components of the mitochondrial TCA cycle, promoting lipoylated protein aggregation followed by a decrease in iron-sulfur cluster proteins, thereby inducing proteotoxic stress, leading to cell death. ES, elesclomol; FDX1, ferredoxin 1; DLAT, dihydrolipoyl transacetylase; TCA, tricarboxylic acid cycle.

The exact regulatory mechanism of copper induced cell death still needs further elucidation, although various pathways have been proposed, including induction of apoptosis, induction of reactive oxygen species, inhibition of the ubiquitin proteasome system. Currently in the study of Tsvetkov et al. (15), it was demonstrated that the mechanism of cuproptosis involves a copper ionophore named “elesclomol”, which can bind Cu^2+^ in the extracellular environment and transport Cu^2+^ into the cells. Intracellularly, several critical genes are involved in this process. *FDX1* gene encodes a reductase ferredoxin 1 reducing Cu^2+^ to Cu^+^. *DLAT* gene encodes an enzyme called dihydrolipoyl transacetylase that is a part of pyruvate dehydrogenase involved in the tricarboxylic acid cycle and is a protein target for lipidation. FDX1 is a key regulator of cuproptosis and an upstream regulator of protein lipoylation. On the one hand, FDX1 promotes lipoylation of pyruvate dehydrogenase, and Cu^2+^ directly binds to lipoylated proteins (mainly DLAT), followed by aberrant oligomerization of DLAT, resulting in tricarboxylic acid cycle (TCA) inhibition, and on the other hand, FDX1 reduces Cu^2+^ to Cu^+^, leading to inhibition of iron sulfur protein clusters synthesis, disruption of iron sulfur protein clusters stability, and ultimately leads to proteotoxic stress and cell death (**Figure 3**).

Cancer cells can evade the regulated cell death pathway, which is also one of the important hallmarks of cancer. Currently, one of the major challenges in cancer therapies is the escape of cancer cells from cell death pathways. The discovery of cuproptosis will provide a novel target to overcome the resistance of cancer cells for cell death. Existing evidence indicates that the Cu^2+^ carrier, elesclomol, can kill specific drug-resistant cancer cells (125). In recent studies on lower grade gliomas (LGG), arguing that copper death may serve as a potential therapeutic target for LGG (126). Researches have shown that cuproptosis related genes may play a great role in the diagnosis and prognosis of some types of cancer. In the research of pancreatic adenocarcinoma (PAAD) by Huang et al., the cuproptosis-related gene index (CRGI) was developed through machine algorithm, and its immunological characteristics were studied by exploring its impact on the expression of immune checkpoints, prospective immunotherapeutic response, etc. A new CRGI was identified and verified, and the cuproptosis-related gene was found to be a reliable diagnostic biomarker in PAAD (127). Sha et al., in a study of triple-negative breast cancer (TNBC), identified two clusters of cuproptosis related genes (CRG) with features of immune cell infiltration and demonstrated that the CRG signature may be used to assess tumor immune cell infiltration, clinical features, and prognostic status. Their study has shown the potential effect of CRG on the tumor microenvironment (TME), clinicopathological characteristics, and prognosis of TNBC which are potential tools for predicting patient outcomes (128). In the study of clear cell renal cell carcinoma (ccRCC), Wang et al. found that ccRCC samples had significantly lower FDX1 expression levels than normal tissue samples and lower FDX1 gene expression levels were strongly associated with higher cancer grades and more advanced tumor-node-metastasis stages. The results of multivariate and univariate analyses indicated that ccRCC patients with low FDX1 expression had shorter overall survival (OS) than those with high FDX1 expression. The study illustrates that in ccRCC, reduced FDX1 expression is associated with disease progression, poor prognosis and dysregulated immune cell infiltration which illustrates that the cuproptosis related gene may serve as a potential prognostic predictor for ccRCC patients and may provide new insights into cancer treatment (129, 130). Zhu et al. performed a comprehensive analysis of cuproptosis related molecular patterns in 1274 colorectal cancer specimens based on 16 cuproptosis regulators and revealed a novel cuproptosis related molecular pattern associated with TME phenotypes, and the formation of a cuproptosis score will further enhance the understanding of TME characteristics and instruct a more personalized immunotherapy schedule in colorectal cancer (131). Lv et al. explored the association of cuproptosis related genes with skin cutaneous melanoma (SKCM) prognosis by accessing and analyzing a public database, and found that 11 out 12 genes were upregulated in melanoma tissues and three genes (*LIPT1, PDHA1, and SLC31A1*) were of predictive value for melanoma prognosis. Further exploration found that *LIPT1* expression was increased in melanoma biopsies and was an independent favorable prognostic indicator for melanoma patients (132). Zhang et al. integrated a set of bioinformatics tools to analyze the expression and prognostic significance of FDX1, a key regulator of cuproptosis. The cuproptosis related risk score (CRRS) was derived by correlation analysis. The metabolic features, mutation signatures, and immune profile of CRRS-classified hepatocellular carcinoma (HCC) patients were investigated, and the role of CRRS in treatment guidance was analyzed. FDX1 was found to be significantly downregulated in HCC and its high expression was associated with longer survival time. HCC patients in the high CRRS group had significantly worse OS and enriched in tumor related pathways. Mutational analysis revealed that several tumor suppressors such as tumor protein P53 (TP53) and Breast cancer susceptibility gene 1 (BRCA1) -associated protein 1 (BAP1) were mutated at a higher frequency in high CRRS HCC patients, illustrating that cuproptosis related signatures are helpful in predicting prognosis and guiding the treatment of HCC patients (133). There are also scientists building a liver cancer prognosis model based on cuproptosis related mRNAs and lncRNAs, which can effectively predict the potential survival of liver cancer patients as well as evaluate the infiltration of immune cells, tumor mutation burden, and sensitivity to antitumor drugs in liver cancer (134). Li et al. systematically evaluated cuproptosis patterns in bladder cancer (BLCA) patients based on 46 cuproptosis related genes and correlated these cuproptosis patterns with TME phenotypes and immunotherapy effects. For the evaluation of individual patients, a cuproptosis risk score (CRS) for prognosis and a cuproptosis signature for precise TME phenotypes and immunotherapy efficacy prediction were constructed. Finally, it was demonstrated that CRS and cuproptosis signature have potential roles in predicting prognosis and immunotherapy efficacy in BLCA (135). A pan-cancer analysis revealed that transcription and protein expression of FDX1 was significantly reduced in most cancer types, and furthermore, FDX1 expression levels were closely correlated with immune cell infiltration, immune checkpoint genes, and immune regulatory genes to some extent. Due to its important role in tumorigenesis and tumor immunity, FDX1 can serve as a potential therapeutic target and prognostic marker in various malignancies (136–138). Altogether, putting the discovery of cuproptosis may provide a new strategy for cancer prognosis as well as treatment.

## 5 Conclusion and perspective

Copper plays an irreplaceable role as a micronutrient in the human body, and both deficiency or overload of copper in the body can negatively affect the human body, therefore, the mechanism of copper metabolism in cells keeps copper at a stable level to achieve copper homeostasis. Copper metabolism is also closely associated with cancer development, and copper is able to affect cancer cell proliferation and metastasis. Intracellular copper has a great connection with cancer, therefore, targeting copper in cancer therapy may play a great role. Currently, using copper complexes for cancer treatment, copper chelators with copper ionophores are two good choices, but the efficacy of copper chelators alone is not significant, and copper ionophores are still in the development stage, which also lack selectivity for targeting cancer cells. Therefore, improving selectivity against cancer cells is a worthy goal of investigation in the future. In a recent study on cell death, a novel concept cuproptosis, which is a copper dependent cell death induced by copper, was proposed, and the exact regulatory mechanism of this novel regulated cell death still needs to be continued to be explored. The proposal of cuproptosis presents a new avenue for the treatment of cancer.

It has a link between copper metabolism and cuproptosis. The dysregulation of copper metabolism in the body, such as copper overload may lead to cuproptosis. In addition, cuproptosis also offer a novel strategy for targeted cancer therapy. Copper metabolism and cuproptosis is worthy of further exploration and application to conquer cancer in clinic.

## Author contributions

Conceptualization, JL, YL and JW; investigation, ZW, DJ and SZ; writing—original draft preparation, ZW, DJ and YJ; writing—review and editing, PA, JL, YL and JW; Visualization, ZW, ND and JL; supervision, JL, YL and JW; project administration, YL and JW; funding acquisition, YL and JL. All authors have read and agreed to the published version of the manuscript.

## Glossary

**Table:**

ROS	reactive oxygen species
WD	Wilson disease
MD	Menkes disease
Ctr1	copper transporter receptor 1
Ctr2	copper transporter receptor 2
DMT1	divalent metal transporter 1
ATP7A	copper-transporting ATPase 1
ATP7B	copper-transporting ATPase 2
MT	metallothionein
GSH	glutathione
CCS	copper chaperone for superoxide dismutase
Atox1	antioxidant protein 1
Cox	cytochrome c oxidase
Cox17	cytochrome c oxidase copper chaperone 17
SOD1	superoxide dismutase 1
CuPrs	cuproproteins
MAPK	mitogen activated protease kinase
HIF-1	hypoxia inducible factor-1
bFGF	basic fibroblast growth factor
TNF-α	tumor necrosis factor alpha
EMT	epithelial to mesenchymal transition
LOX	lysine oxidase
ECM	extracellular matrix
TTM	tetrathiomolybdate
D-Pen	D-penicillamine
DSF	disulfiram
ACC	adrenocortical carcinoma
BRCA	breast invasive carcinoma
LGG	lower grade glioma
MESO	mesothelioma
SKCM	skin cutaneous melanoma
ES	elesclomol
FDX1	ferredoxin 1
DLAT	dihydrolipoyl transacetylase
TCA	tricarboxylic acid cycle
PAAD	pancreatic adenocarcinoma
TNBC	triple-negative breast cancer
HCC	hepatocellular carcinoma
ccRCC	clear cell renal cell carcinoma
BLCA	bladder cancer
TME	tumor microenvironment
TP53	tumor protein P53
BRCA1	breast cancer susceptibility gene 1
OS	overall survival
CRG	cuproptosis related genes
CRS	cuproptosis risk score
CRRS	cuproptosis related risk score
CRGI	cuproptosis-related gene index.
